# Complete thoracoscopic enucleation of giant leiomyoma of the esophagus: a case report and review of the literature

**DOI:** 10.1186/1749-8090-9-34

**Published:** 2014-02-14

**Authors:** XiaoXing Hu, Hui Lee

**Affiliations:** 1 Department of Thoracic Surgery, Beijing Chao-Yang Hospital, Beijing, China; 2 Department of Thoracic Surgery, Beijing Key Laboratory of Respiratory and Circulation, Beijing Chao-Yang Hospital, Beijing Institute of Respiratory Medicine Capital Medical University, Beijing, China

**Keywords:** Giant esophageal leiomyoma, Complete thoracoscopic surgery, Enucleation

## Abstract

Esophageal leiomyoma is one of the most common types of benign esophagus tumors. Giant leiomyoma of the esophagus is traditionally treated by open thoracotomy, which has large incision. We report a case of complete thoracoscopic enucleation of giant leiomyoma in a chinese patient.

## Background

Esophageal leiomyoma is one of the most common types of benign esophagus tumors [1]. Giant leiomyoma (GLM) of the esophagus is defined as esophageal leiomyoma with a diameter larger than 10 cm [2,3]. Giant leiomyoma of the esophagus is traditionally treated by open thoracotomy, which causes considerable operative trauma and negatively affects respiratory function and resumption of a normal diet. In recent years, with the rapid development of minimally invasive techniques, endoscopy has been used for the treatment of giant leiomyoma of the esophagus. This case report describes a patient with giant leiomyoma of the middle and lower esophagus who underwent thoracoscopic enucleation at our hospital.

## Case presentation

**Figure 1:**
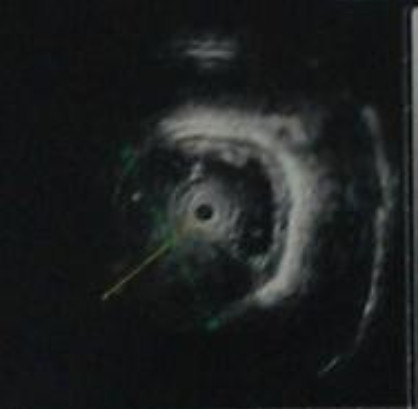
Ultrasonic gastroscopy revealed a giant mucous prominence of esophagus at 23 cm from the incisors and it originated from muscularis propria.

**Figure 2:**
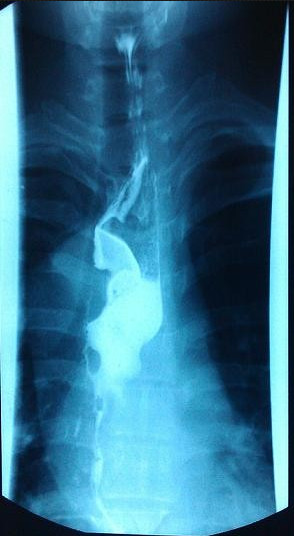
Upper gastrointestinal contrast radiography showed a leiomyoma of middle esophagus and compensatory expansion of the upper esophagus.

**Figure 3:**
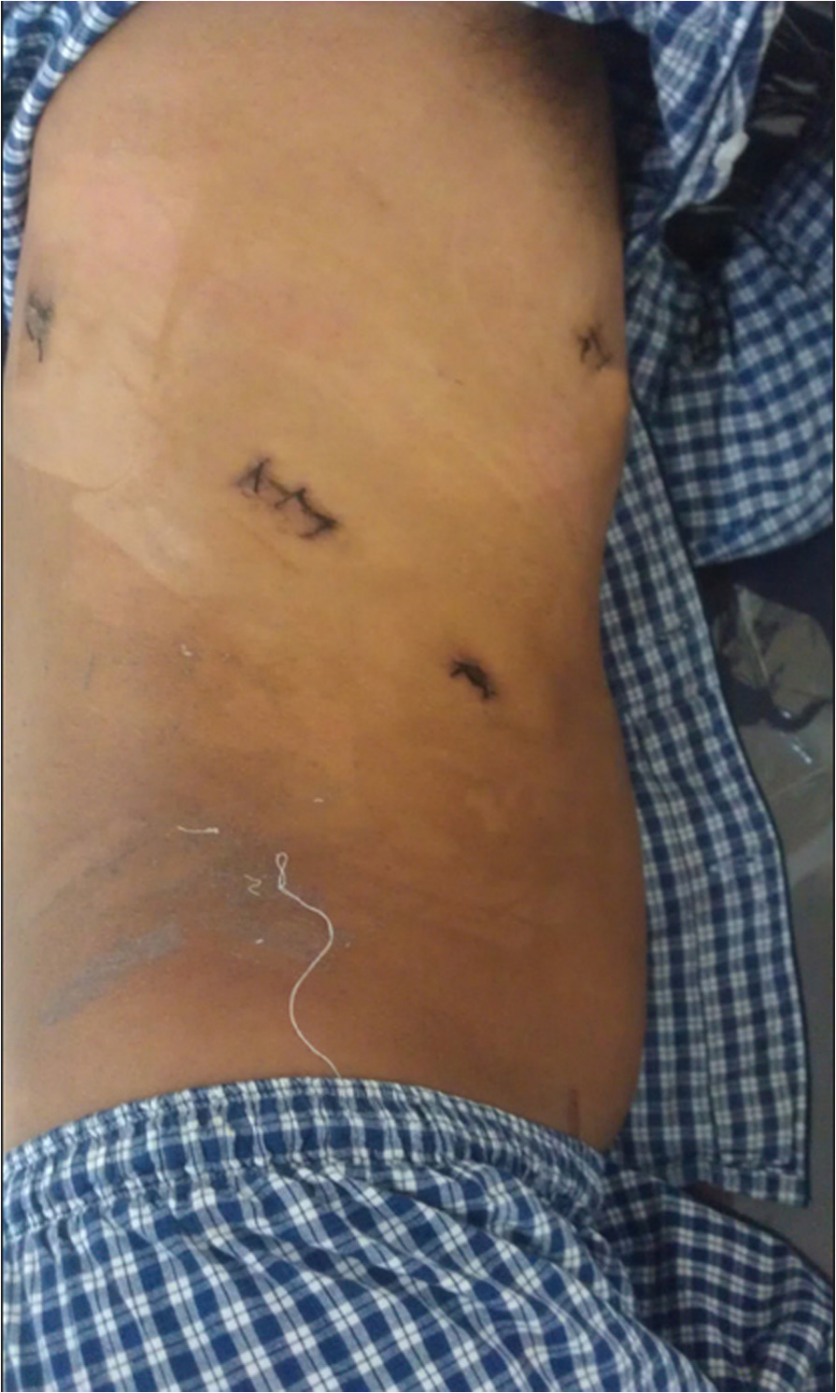
Incision (postoperatively).

**Figure 4:**
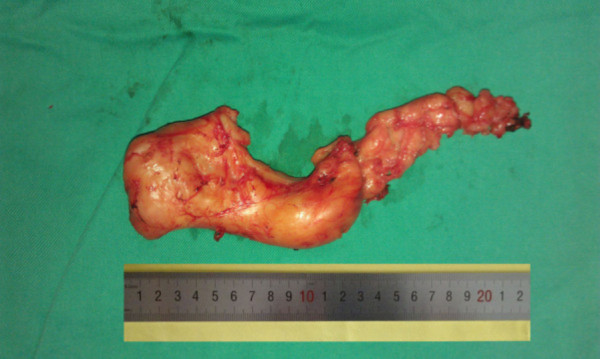
Operative specimen.

**Figure 5:**
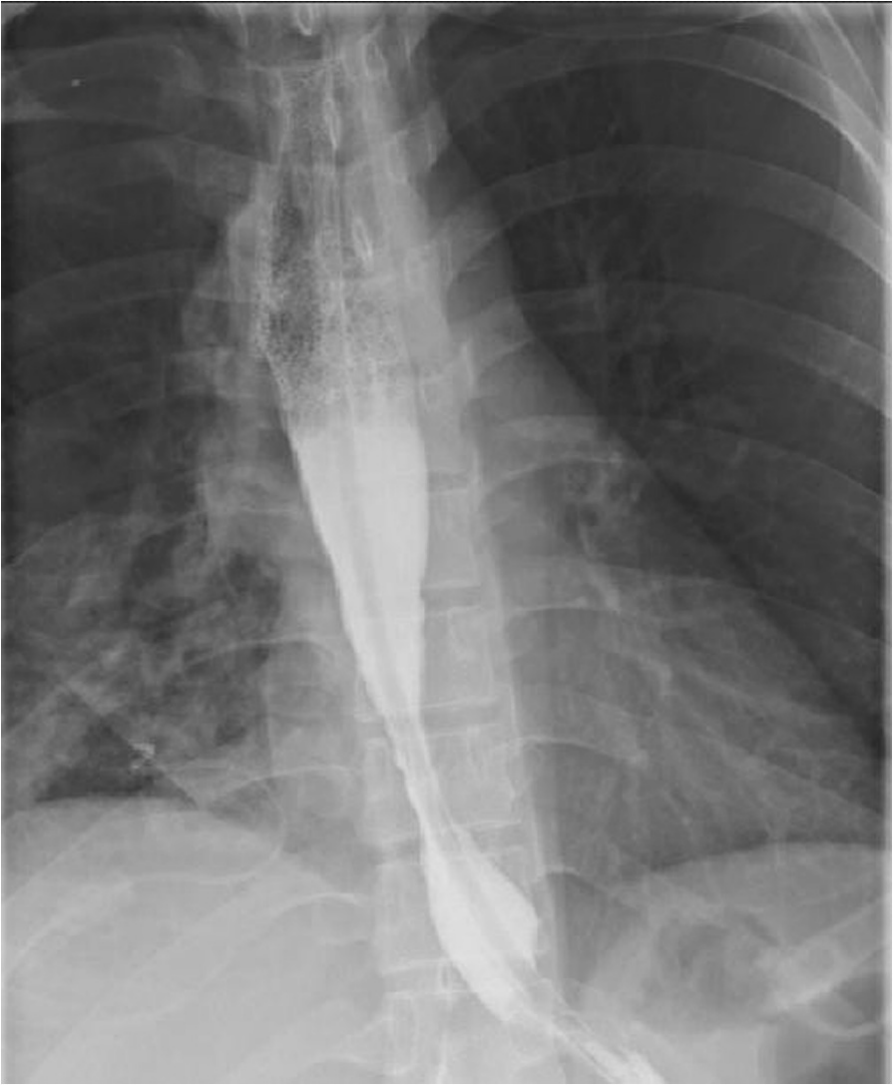
Postoperative contrast radiography of esophagus before recovery to normal diet.

A 26-year-old chinese man was admitted to our hospital because of dysphagia for over one year. Results of his physical examination were normal. Gastroscopy revealed a giant esophageal submucosal protrusion with a smooth surface that was located 20 to 26 cm from the incisors. Ultrasonic gastroscopy revealed a giant mucous prominence of the esophagus located 23 cm from the incisors that originated from the muscularis propria and was suspected to be a leiomyoma because of its smooth surface and normal color (Figure 1). Chest computed tomography (CT) showed a round high-density shadow on the right front edge of the esophagus at the level of the aortic arch to the inferior pulmonary vein, and this was suspected to be a leiomyoma of the middle esophagus. Upper gastrointestinal contrast radiography showed a leiomyoma of the middle esophagus and compensatory expansion of the upper esophagus (Figure 2). After preoperative preparation, surgery was performed under single-lumen endotracheal intubation. The patient was placed in the left lateral position. An incision was made in the eighth intercostal space at the right posterior axillary line, and a thoracoscope was introduced through the incision. For the operation, one incision was made in the seventh intercostal space between the right scapular line and the posterior axillary line, and another incision was made in the fourth intercostal space between the auscultatory triangle and the anterior axillary line (Figure 3). Exploration revealed that the tumor had spread to the entire length of the thoracic esophagus. The pleura mediastinalis was incised above the diaphragm to expose the lower segment of the esophagus. The muscularis of the esophagus was cut to expose the capsule of the tumor, and a well-defined solid tumor with an intact capsule and rich blood supply was revealed. The lower pole of the tumor along the capsule was gradually isolated. The lower pole of the tumor was sutured with thick silk to facilitate pulling up of the tumor. The incision in the pleura mediastinalis and the muscularis of the esophagus was extended in the direction of the cranium. The arch of the azygos vein was severed using an Endo GIA Universal Stapler. The incision was extended until it reached the upper edge of the tumor, allowing for exploration of the esophageal mucosa and gradual isolation of the tumor outside the mucosa until complete tumor resection was achieved. Two mucosal ruptures (with diameters of about 0.4 cm and 0.3 cm) in the middle esophagus occurred because of the tight adhesion of the tumor to the mucosa. Both ruptures were repaired by interrupted suturing with absorbable thread. Observation with an esophagoscope introduced through the mouth showed that the esophageal mucosa was intact, and no bubbles were observed after gas injection. The interrupted sutures in the muscularis of the esophagus were embedded in the wound. The operation took approximately 180 min to complete. The tumor was approximately 22.5 × 10 × 7.5 cm^3^ in size and tough in quality, with a lobulated ellipsoidal shape (Figure 4). After the operation, the patient underwent water fasting, gastrointestinal decompression, acid suppression, and anti-infection treatment. The patient’s exhaustion abated and defecation resumed on the fifth postoperative day. Contrast radiography of the esophagus on the sixth postoperative day showed intact smooth esophageal mucosa with no obvious leakage (Figure 5), so the stomach tube was removed and the patient began to consume liquid food. Postoperative pathologic results showed a spindle-cell tumor of the esophagus without significant cellular atypia with 0–1 karyokinesis/10 high-power fields, supporting the diagnosis of esophageal leiomyoma. The patient was discharged on the 10th postoperative day. He has been followed up for 10 months, and all his symptoms have disappeared with no abnormalities.

### Discussion

Esophageal leiomyoma is more common than other esophageal benign tumors [1]. Its biological origin is the muscularis of the esophagus, and it is most commonly located in the middle or lower segments of the esophagus [4]. According to the literature, the size of esophageal leiomyomas vary from 1 cm to 29 cm in diameter [2]. Patients with esophageal leiomyoma often have no specific symptoms, and they are diagnosed during routine physical examinations. Esophageal leiomyomas greater than 10 cm in diameter are generally designated as giant leiomyomas [2,3]. Most patients with giant leiomyoma of the esophagus have symptoms such as dysphagia and chest congestion. The traditional surgical approach for esophageal leiomyoma is typically referred to as “small surgery, large incision” as it involves open thoracotomy, and the tumor may even be resected through a thoracoabdominal incision in combination with gastroesophagostomy or tumor enucleation [3,5]. Because of the large tumor size and unclear boundaries with surrounding tissues, all previously reported giant leiomyomas of the esophagus [2,3,5-10] were treated by open thoracotomy or tumor resection through a thoracoabdominal incision in combination with gastroesophagostomy. Traditional open thoracotomy can cause relatively serious operative trauma and negatively affects postoperative respiratory function and diet restoration, in addition to inevitable anastomotic complications [3,6,11], all of which can worsen the prognosis. In recent years, a series of domestic and international centers have gradually implemented minimally invasive surgeries for the treatment of esophageal leiomyoma, including resection or enucleation [12-18] of esophageal leiomyoma by thoracoscopy [1,4,11], laparoscopy [1,4], or Da Vinci robot-assisted thoracoscopy [19]. All of these surgical approaches achieved good curative effects, and their complications were less than with open thoracotomy. A report by Ozdil et al. [20] described treatment of esophageal leiomyoma with endoscopic percutaneous injection of ethanol. However, most of the esophageal leiomyomas treated in this manner were smaller than 10 cm. There are fewer reports describing minimally invasive surgeries for giant leiomyomas of the esophagus. Chen [16] reported two cases of giant leiomyoma treated by thoracoscopic enucleation, but the tumor sizes were 10 × 7 × 4 cm and 8 × 6 × 3 cm, which are smaller than giant leiomyoma of the esophagus. In this report, a patient with giant leiomyoma of the esophagus, 22.5 × 10 × 7.5 cm in size, which is the largest reported in current literature on PubMed and Cochrane, underwent thoracoscopic enucleation at our hospital. He received detailed preoperative assessment, and the entire surgery was conducted using a thoracoscope. The patient resumed intake of a normal diet after the operation. After following the patient for 8 months after the operation, it was determined that a curative effect was achieved. Examination of the integrity of esophageal mucosa by gastroscopy at the end of the operation is crucial for resuming normal diet postoperatively, as well as avoiding esophageal fistulas and related complications [18]. Laceration of the esophageal mucosa is the most common intraoperative and postoperative complication associated with enucleation of esophageal leiomyoma [15]. When the mucosal layer tightly adheres to the muscularis of the esophagus, lacerations of different sizes and numbers can easily occur during the process of tumor isolation. The lacerated mucosa sites should immediately be repaired by suturing with absorbable thread under thoracoscopic guidance followed by interrupted suturing of the muscularis. The repair work and the integrity of esophageal mucosa should be confirmed with gastroscopy.

## Conclusion

In conclusion, thoracoscopic enucleation of giant leiomyomas of the esophagus does not only result in completely resection the tumor to avoid its enlargement and malignant transformation, but also maximally preserves the integrity of esophageal mucosal structures. This approach is feasible and effective in the surgical treatment of giant leiomyomas of the esophagus.

## Consent

Written informed consent was obtained from the patient for publication of this case report and any accompanying images. A copy of the written consent is available for review by the Editor-in-Chief of this journal.

## Abbreviations

VATS: Video-assisted thoracoscopic surgery; CT: Computerized tomography; GLM: Giant leiomyoma.

## Competing interests

The authors declare that they have no competing interests.

## Authors’ contributions

XH performed surgical management of this case, wrote the manuscript and arranged follow-up (First Author). HL supervised XH to write this manuscript and gave comments to revise it (Corresponding author). Both authors read and approved the final manuscript.
